# Analysis of Peptidases in Non-Infected and *Trypanosoma cruzi*-Infected Mouse Embryo Hepatocyte Cells

**Published:** 2008-06

**Authors:** Ana Cristina Nogueira de Melo, André Luis Souza dos Santos, Maria Nazareth Leal Meirelles, Marta Helena Branquinha, Alane Beatriz Vermelho

**Affiliations:** 1 *Departamento de Microbiologia Geral, Instituto de Microbiologia Prof Paulo de Góes (IMPPG), Centro de Ciências da Saúde (CCS), Bloco I, Universidade Federal do Rio de Janeiro (UFRJ), Ilha do Fundão, Rio de Janeiro, RJ, Brazil;*; 2 *Laboratório de Ultraestrutura Celular, Departamento de Ultraestrutura e Biologia Celular, Instituto Oswaldo Cruz, FIOCRUZ, Avenida Brasil 4365, Rio de Janeiro, RJ, Brazil*

**Keywords:** hepatocytes, peptidases, cruzipain, metallopeptidase, *Trypanosoma cruzi*

## Abstract

Cellular and extracellular peptidase profiles from non-infected and *Trypanosoma cruzi*-infected hepatocyte cell cultures were characterized by sodium dodecyl sulfate-polyacrylamide gel electrophoresis (SDS-PAGE) containing different copolymerized proteins as substrates. A 100 kDa metallopeptidase activity was detected in the cellular extracts and in the culture supernatant fluids of both systems, had the ability to exclusively degrade gelatin. However, non-infected hepatocytes produced an additional extracellular metallopeptidase of 85 kDa. In the non-infected and in the infected hepatocytes, a cysteine peptidase migrating in gelatin-SDS-PAGE at 60 kDa presented the broadest specificity, since it was also able to hydrolyze casein and hemoglobin. The 100 kDa component was only detected at alkaline pH and predominantly expressed in non-infected hepatocytes. Conversely, the 60 kDa cysteine peptidase was only observed in acidic condition and its production was robustly augmented in *T. cruzi*-infected cells, probably due to the cysteine peptidase synthesized by the parasites, as corroborated by immunoblotting assay using anti-cruzipain antibody. Collectively, these results suggest that peptidases may be involved in the interaction process between *T. cruzi* and hepatocytes *in vitro*.

## INTRODUCTION

*Trypanosoma cruzi*, the etiological agent of Chagas’ disease, is an intracellular protozoan parasite able to invade and multiply inside a wide variety of mammalian tissues and cells. *T. cruzi* employs multiple molecular strategies to invade a broad range of nonphagocytic cells (1); One of which is the expression of peptidases that play relevant roles in different phases of the parasite-host relationship. In this context, several proteolytic enzymes have been extensively studied in *T. cruzi*, including cysteine-, serine-, threonine- and metallo-type peptidase classes (2, 3). The most abundant and the best characterized among these enzymes is cruzipain, a cysteine peptidase expressed as a complex mixture of isoforms by all developmental stages of the parasite, including some membrane-bound isoforms. The enzyme is an immunodominant antigen in human chronic Chagas’ disease and seems to be crucial for parasite growth, development and tissue/host cell penetration. For these reasons, cruzipain has been proposed as a therapeutic target for treatment of Chagas’ disease (reviewed in 2).

During intracellular development of *T. cruzi*, the expression of extracellular matrix components was demonstrated in the evolution of the fibrosis process in a murine model (4). For this reason, the role of peptidases capable of degrading components of the extracellular matrix, such as matrix metallopeptidases (MMPs), during the *T. cruzi* invasion process as well as in the regulation of pathogenic conditions seems to be of extreme significance. In this context, our laboratory detected the active (85 kDa) and latent (100 kDa) form of MMP-9, using a rabbit anti-mouse MMP-9 antibody in western blotting and immunocyto-chemistry analyses in *T. cruzi*-infected hepatocyte cultures. Conversely, only the latent form of MMP-9 was detected in non-infected hepatocytes (5). In the present work, we have evaluated the ability of the cellular and extracellular peptidases, detected in the non-infected and in *T. cruzi*-infected primary cultures of mouse embryo hepatocytes, in degrading different protein substrates. In addition, we looked for cruzipain molecules during the *in vitro* interaction.

## MATERIALS AND METHODS

### Hepatocyte culture

Hepatocytes were isolated from mouse embryo livers as previously described with some adaptations (6, 7). Briefly, ten mouse embryo livers were asseptically removed and washed with Hank’s balanced salt solution Ca^+2^ and Mg^+2^ free (HBSS-CMF). Longitudinal cuts were made in each lobe for enzyme access and the livers were then incubated at 37°C for 20 min with 0.05% collagenase (Type II Wortington) in approximately 50 ml HBSS-CMF. The organs were dispersed by pipetting, and liver cells were collected by centrifugation at 200 g for 3 min. Viable cells were purified by sedimentation at room temperature for 10 min with MEM/199 medium containing 10% fetal calf serum (FCS). Cells yields were ~2 × 10^7^ ml (total 10 ml) and viability greater than 90%, as assessed by the trypan blue exclusion methodology. Cells were seeded on tissue culture flasks. Four hours later fresh defined medium [MEM/199 supplemented with 5 mM CaCl_2_, 10 mg/ml insulin, transferrin and selenium (ITS), 1 ng/ml glucagon, 50 ng/ml epidermal growth factor (EGF), 3.5 × 10^-6^ M hydrocortisone and 1 ng/ml bovine serum albumin (BSA)] was added and replaced daily. Cultures were incubated at 37°C in a humidified atmosphere containing 5% CO_2_.

### Parasite

Bloodstream trypomastigote forms of *T. cruzi* Y strain were obtained from seven days-infected mice at the peak of parasitemia. Blood was collected with 3.8% sodium citrate as an anticoagulant agent, and centrifuged at 150 g for 10 min. The tube was then kept at 37°C for 30 min, allowing trypomastigotes to move from the pellet to the supernatant. The pellet was discarded and the parasite containing supernatant was collected, centrifuged at 900 *g* for 10 min and washed with cold PBS (150 mM NaCl; 20 mM phosphate buffer, pH7.2) (8). Cell number was estimated by counting the parasites in a Neubauer chamber. Amastigote forms were obtained from the supernatant of Vero cell cultures infected with bloodstream trypomastigotes as previously described by Souto-Padrón *et al*. (9).

### Parasite-hepatocyte interaction

Hepatocyte primary cultures were infected 48 h after plating with 5 × 10^6^ parasites per flask. Cultures were incubated for 48 h at 37°C in a humidified atmosphere containing 5% CO_2_. A four day old non-infected hepatocyte culture was used as a control system (5, 7).

### Hepatocyte extracts

Cells were scraped from flasks, centrifuged at 2000 *g* for 20 min at 4°C, washed three times with cold PBS, and lysed at room temperature by addition of 100 μl SDS-PAGE sample buffer (125 mM Tris, pH6.8; 4% SDS; 20% glycerol; 0.002% bromophenol blue). Alternatively non-infected and the *T. cruzi*-infected hepatocyte culture supernatants were collected, and passed over a 0.22-μm filtration unit (Millipore). Samples were concentrated by dialysis (cut-off 9000 Da) against polyethyleneglycol 4000 overnight at 4°C. Protein concentrations were determined by the method described by Lowry *et al*. (10), using BSA as standard. Finally, the SDS-PAGE sample buffer was added in a 7:3 (supernatant:sample buffer) proportion.

### Substrate-gel electrophoresis

Proteolytic activities in culture supernatants (50 μg protein) and in cellular extracts (1 × 10^6^ hepatocytes) were evaluated by 10% SDS-PAGE containing 0.1% gelatin, casein or hemoglobin as substrates incorporated into the gel (11). After electrophoresis at a constant voltage of 160 V at 4°C, the gels were soaked for 1 h at 25°C in 2.5 % Triton X-100. Gels were then incubated for 24 h at 37°C in 50 mM sodium phosphate buffer, pH5.5, supplemented with 2 mM dithiothreitol (DTT) in the absence or in the presence of 10 μM E-64, and in 50 mM glycine-NaOH buffer, pH 10, in the absence or in the presence of 10 mM 1,10-phenanthroline. The gels were stained for 1 h with 0.2% Coomassie brilliant blue R-250 in methanol-acetic acid-water (50:10:40) and destained in the same solvent (12). Molecular masses of the peptidases were calculated by comparison of the mobility of GIBCO BRL SDS-PAGE standards (Grand Island, NY, USA). The gels were dried, scanned and the densitometric analysis was performed with the use of the Kodak Digital Science EDAS 120 software as described by Elias *et al*. (12). In these analyses, bands in each gel were manually selected using the free selection tool provided by the software. Band areas were then determined by repeating this process three times, to diminish the probability of errors in these estimations. Values of band area were further integrated with means of gray level in selected bands, generating densitometric values that were used in the comparison between corresponding bands from the different gels.

### Western-blotting

Cellular protein extracts (equivalent to 100 μg protein) from non-infected and *T. cruzi*-infected hepatocytes as well as amastigote forms of *T. cruzi* Y strain (50 μg), the intracellular replicative stage observed during mammalian infection, were electrophoretically transferred (100 V/300 mA for 2 h at 4°C) to a nitrocellulose membrane. The membrane was blocked in 10% nonfat dried milk in a blocking solution (150 mM NaCl; 10 mM Tris, pH7.5; 0.05% Tween 20) for 1 h at room temperature under constant agitation and washed three times (10 min per wash) in the same solution. The membrane was incubated with anti-cruzipain polyclonal antibody kindly provided by Dr. Juan-Jose Cazzulo (Instituto de Investigaciones Biotecnologicas, Universidad Nacional de General San Martin, Buenos Aires, Argentina) at 1:100 dilution for 2 h and then washed as described above. Finally, the blot was incubated with the secondary antibody for 1 h, followed by immunodetection by chemiluminescence (ECL reagent).

## RESULTS AND DISCUSSION

**Figure 1:**
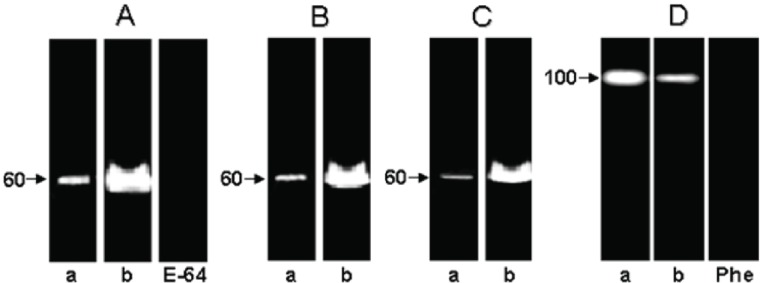
Effect of different protein substrates [gelatin (A, D), casein (B) and hemoglobin (C)] incorporated into 10% SDS-PAGE on the detection of cellular peptidases in the non-infected (a) and in the *T. cruzi*-infected (b) mouse embryo hepatocyte cell cultures. Gel strips (A-C) containing extracts from 1 × 10^6^ hepatocytes were incubated at 37°C for 24 h in 50 mM sodium phosphate buffer, pH 5.5, supplemented with 2 mM DTT in the absence (a, b) or in the presence of 10 μM E-64. The second set of gelatin-gel strips (D) was incubated in 50 mM glycine-NaOH buffer, pH 10, in the absence (a, b) or in the presence of 10 mM 1,10-phenanthroline (Phe). Molecular masses of the peptidases, expressed in kilodalton, are indicated in the left side of each panel.

**Figure 2:**
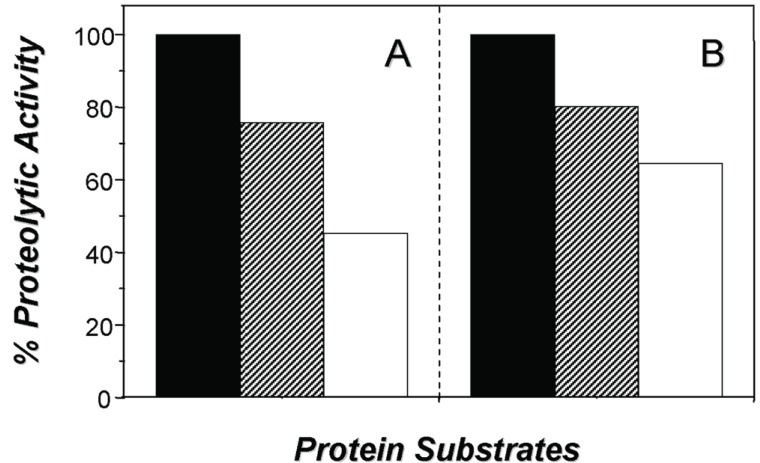
Percentage of the 60 kDa cysteine proteolytic enzyme activity detected in the non-infected (A) and in the *T. cruzi*-infected hepatocytes (B), after SDS-PAGE using gelatin (black bars), casein (hachured bars) and hemoglobin (white bars) as substrate incorporated into the gels. The gels were densitometrically analyzed, and each bar represents the value of three independent measurements.

*Trypanosoma cruzi* has a complex life cycle comprising different developmental stages, which permits its adaptation to very diverse host environments. The tropism of *T. cruzi* for different tissues, including liver, has been reported (5, 7, 13). A recent study showed invasion and development through the entire *T. cruzi* cell cycle in an *in vitro* system of primary cultures of mouse embryo hepatocytes (7), affecting the hepatocyte peptidase production (5). In the present report, the hydrolytic ability of the peptidases present in these culture systems, infected or not with *T. cruzi*, was evaluated by SDS-PAGE containing different proteinaceous substrates. The zymogram gels showed a cell-associated peptidase migrating at 60 kDa in gelatin-SDS-PAGE. This proteolytic activity was detected in the control and infected hepatocyte cellular extracts only at acidic pH value (5.5) and in the presence of a reducing agent (DTT) (Fig. 1A, lanes a,b), being its hydrolytic activity completely abolished by 10 μM E-64, a potent cysteine peptidase inhibitor (Fig. 1, lane E-64). The 60 kDa cysteine peptidase presented the broadest specificity, since it was also able to degrade casein (Fig. 1B) and hemoglobin (Fig. 1C), although significantly less (Figs. 1 and 2). Table 1 summarizes the densitometric measurements of the proteolytic activities expressed in both hepatocyte systems using gelatin as substrate. Additionally, we observed that the activity of the 60 kDa cysteine peptidase was significantly enhanced (approximately 232%) in the *T. cruzi*-infected system (compare lanes a and b in the Fig. 1, and Table 1).

**Table 1:** Densitometric analyses of the gelatinolytic activities detected during the *T. cruzi*-hepatocyte interaction

Molecular mass (kDa)	Peptidase class	(%) Quantitative peptidase expression^a^

60	Cellular cysteine peptidase	232
100	Cellular metallopeptidase	65
85	Extracellular metallopeptidase	0
100	Extracellular metallopeptidase	59

a Variation of the peptidase expression in *T. cruzi*-infected hepatocyte cells, taking expression in non-infected hepatocytes as reference values (100%). The percentage values represent means of three independent measurements.

**Figure 3:**
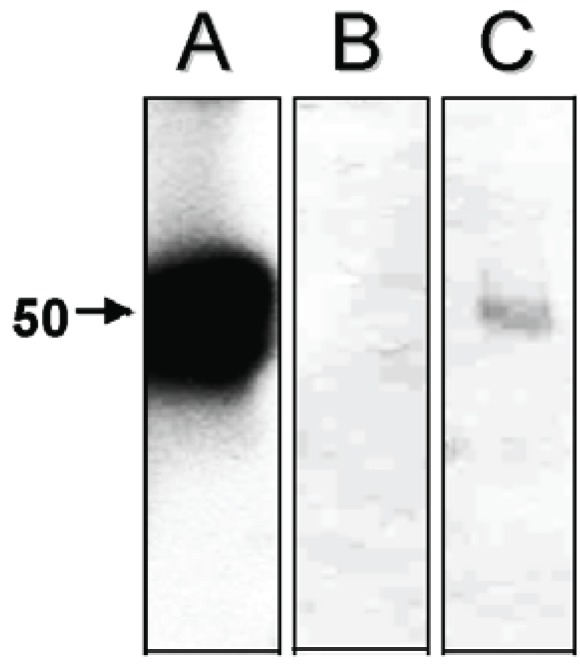
Western blotting showing the cruzipain enzyme detected in the *T. cruzi*-infected hepatocyte cells (C) when compared to non-infected ones (B) and *T. cruzi* amastigote developmental stage (A). Molecular mass of the reactive polypeptide, expressed in kilodalton, is indicated in the left.

Previous studies showed that cathepsin L is the most active cysteine peptidase in hepatocytes, contributing to more than 40% of their total proteolytic activity, being able to hydrolyze several protein substrates (14). However, we cannot discard the hypothesis that the augment in the cysteine peptidase activity in the *T. cruzi*-infected hepatocytes was due to expression of the parasite cysteine peptidase named cruzipain, which migrates in gelatin-SDS-PAGE in this same molecular mass range and belongs to the papain C1 family of cysteine peptidases, with a specificity intermediate between those of cathepsin L and cathepsin B (2). Supporting our idea, western blot analysis evidenced a faint polypeptide band with apparent molecular mass of 50 kDa in the *T. cruzi*-infected hepatocyte cellular extracts after probing with anti-cruzipain polyclonal antibody (Fig. 3, lane C). Conversely, no reactivity was observed when only hepatocyte cells were analyzed (Fig. 3, lane B), demonstrating an absence of immunological cross-reactivity between cysteine peptidases synthesized by the hepatocytes and the parasite.

Corroborating our findings, earlier studies have shown that cruzipain is able to degrade several substrates including gelatin, hemoglobin and casein (15), and this fact may have some positive effect either on the parasite development, penetration and survival inside the host cells (1-3). In previous studies, cruzipain activity has been associated with the growth and differentiation of epimastigotes and amastigotes (2). In order to survive, intracellular organisms must escape the anti-microbial mechanisms of distinct host cells. For instance, cruzipain of *T. cruzi* participates in the nutrition of the parasite at the expense of the host and is involved in the escape mechanisms of the parasite from the host’s immune system (1, 2). Cruzipain stimulates potent humoral and cellular immune responses during infection in both humans and mice. Furthermore, cruzipain modulates the host response in favor of the parasite survive. Stempin *et al*. (16) demonstrated that cruzipain decreases NO and IL-12 production from macrophages stimulated by LPS. IL-10, induced by cruzipain in peritoneal cells, is a cytokine that could enhance the alternative activation of macrophages and favor parasite replication. It is possible that IL-10 released by macrophages could deviate the immune system to the T2 response. IL-10 and some microbial antigens are endogenous inhibitors of regulation of IL-12. Then, the balance between anti-inflammatory (IL-10 and TGF-β) and proinflammatory (IL-12) cytokines at the beginning of *T. cruzi* infection could be crucial for the installation of the parasite (16).

Hepatocyte infections with *T. cruzi* have been reported in association with bacteria in immunosuppressed mice (17). Soeiro *et al*. (18) showed that the surface charge of the mouse embryo hepatocytes was affected by *T. cruzi*, showing a decrease in their Zeta potential of approximately 10% during the first 20 h of parasite-cell interaction. *T. cruzi* is able to replicate in the liver, suggesting the parasite capability of inhibiting innate immunity. Since most physiological liver activities are due to hepatocyte cells, any disturbance in the number of healthy cells may be pivotal to organ homeostasis and function. For example, during human or experimental visceral leishmaniasis, there is an increase in the production of hepatic transaminases, indicating hepatocyte lesions (19, 20).

In the current study, a cell-associated peptidase of 100 kDa was also detected in both systems (Fig. 1D), which was totally inhibited by 10 mM 1,10-phenanthroline (Fig. 1D, lane Phe), indicating that this should be a metallopeptidase or metal-activated peptidase. The activity of the 100 kDa metallopeptidase was only detected under alkaline conditions and was down regulated in the *T. cruzi*-infected hepatocytes (Fig. 1D, lane b) when compared with the non-infected ones (Fig. 1D, lane a, and Table 1), showing a clear modulation in the peptidase production along the infective process. When casein and hemoglobin were incorporated into the gel, no proteolytic activity was observed (data not shown).

**Figure 4:**
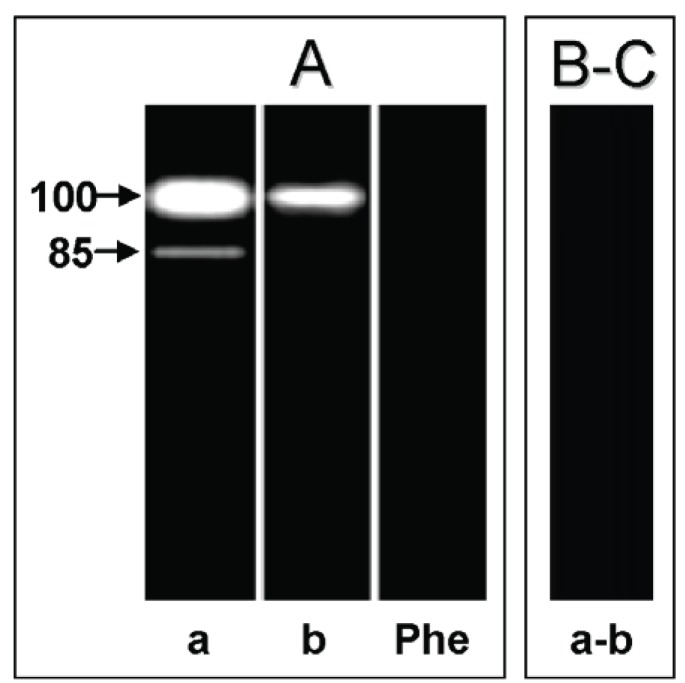
Effect of different protein substrates [gelatin (A), casein (B) and hemoglobin (C)] incorporated into 10% SDS-PAGE on the detection of extracellular peptidases in the non-infected (a) and *T. cruzi*-infected (b) embryo mouse hepatocyte cell cultures. The gel strips containing concentrated supernatant fluids (50 μg) were incubated in 50 mM glycine-NaOH buffer, pH 10, in the absence (**a, b**) or in the presence of 10 mM 1,10-phenanthroline (**Phe**). Molecular masses of the peptidases, expressed in kilodalton, are indicated in the left.

In the cell-free supernatant of both hepatocyte systems, we detected a major metallopeptidase migrating at 100 kDa, active at pH5.5 (data not shown) and pH10 (Fig. 4A, lanes a and b). In the non-infected hepatocyte cell-free supernatant we also detected an additional 85 kDa metallopeptidase only at pH10 (Fig. 4A, lane a), which was also blocked by 10 mM 1,10-phenanthroline (Fig. 4A, lane Phe). Both proteolytic activities were able to degrade only gelatin (Fig. 4B-C). Previous studies have shown by SDS-PAGE the release of gelatin-degrading enzymes migrating at 92 and 95 kDa by human and rat Kupffer cells, respectively (21). These enzymes are named MMP-9 or gelatinase B, and the higher and lower molecular masses of these enzymes correspond to the latent and active forms of MMP-9, respectively (22). Similarly, in a previous work of our research group, we characterized two extracellular metallopeptidases (85 and 100 kDa) as belonging to the MMP-9 family (5). In addition, as visualized in the cellular extract, the 100 kDa extracellular metallopeptidase had its activity significantly reduced in the *T. cruzi*-infected hepatocytes, while the 85 kDa component was not detected (Table 1). The presence of the parasite in the infected hepatocytes may inhibit a biochemical process that culminates in the secretion of these metallopeptidases into the extracellular environment. The fact that the 85 kDa extracellular metallopeptidase, which corresponds to the active form of MMP-9 (5), has been found exclusively in the supernatant fluid of the non-infected hepatocytes, suggesting a role for this enzyme in the breakdown of the ECM in the hepatocyte cell spreading and growth. It is known that MMPs, including MMP-9, participate in a peptidase cascade to remodel the ECM (23). Earlier studies have described an ECM-degrading peptidase in *T. cruzi*, and this proteolytic activity may facilitate invasion of host cells, an activity that is likely to be relevant *in vivo* during the navigation of interstitial tissue spaces by trypomastigote forms (24).

Collectively, the results present herein strongly suggest that expression of cysteine- and metallopeptidase activities is modulated in hepatocyte cells during the *T. cruzi* infection process. In addition, the fact that *T. cruzi* is capable of invasion and development of its entire cell cycle within an *in vitro* system of primary cultures of mouse embryo hepatocytes should make this culture system a useful tool to investigate the pharmacotoxicological effects of new drugs against human Chagas’ disease, since the liver performs numerous important metabolic functions such as detoxification of endogenous and exogenous compounds.

## References

[1] Yoshida N (2006). Molecular basis of mammalian cell invasion by *Trypanosoma cruzi*. An. Acad. Bras. Cienc.

[2] Cazzulo JJ, Stoka V, Turk V (2001). The major cysteine proteinase of *Trypanosoma cruzi*: a valid target for chemotherapy of Chagas’ disease. Curr. Pharmac. Des.

[3] Vermelho AB, Giovanni-De-Simone S, d’Avila-Levy CM, Santos ALS (2007). Trypanosomatidae: peptidases and drugs development. Curr. Enz. Inhib.

[4] Calvet CM, Meuser M, Almeida D, Meirelles MN (2004). *Trypanosoma cruzi*-cardiomyocyte interaction: role of fibronectin in the recognition process and extracellular matrix expression *in vitro* and *in vivo*. Exp. Parasitol.

[5] Nogueira de Melo AC, Meirelles MNL, Porrozzi R (2004). Reduced activity of matrix metalloproteinase-9 in *Trypanosoma cruzi-*infected mouse embryo hepatocyte cell. Hepatol. Res.

[6] Sells MA, Chernoff J, Cerda A, Bowers C (1985). Long-term culture and passage of human fetal liver cells that synthesize albumin *in vitro*. Cell Dev. Biol.

[7] Porrozzi R, Soares R, Meuser M, Guguen-Guillouzo C (1997). Invasion and development of *Trypanosoma cruzi* in primary cultures of mouse embryo hepatocytes. Mem. Inst. Oswaldo. Cruz.

[8] Meirelles MNL, Souto-Padrón T, De Souza W (1984). Participation of the cell surface anionic sites in the interaction between *Trypanosoma cruzi* and macrophages. J. Submicrosc. Cytol.

[9] Souto-Padrón T, Campetella OE, Cazzulo JJ, De Souza W (1990). Cysteine proteinase in *Trypanosoma cruzi*: immunocytochemical localization and involvement in parasite-host cell interaction. J. Cell Sci.

[10] Lowry OH, Rosebrough NJ, Farr AL, Randall RJ (1951). Protein measurement with the Folin phenol reagent. J. Biol. Chem.

[11] Nogueira de Melo AC, d’Avila-Levy CM, Branquinha MH, Vermelho AB (2002). *Crithidia guilhermei*: gelatin- and hemoglobin-degrading extra-cellular metallopeptidases. Exp. Parasitol.

[12] Elias CGR, Pereira FM, Silva BA, Alviano CS (2006). Leishmanolysin (gp63 metallopeptidase)–like activity extracellularly released by *Herpetomonas samuelpessoai*. Parasitology.

[13] Melo RC, Brener Z (1978). Tissue tropism of different *Trypanosoma cruzi* strains. J. Parasitol.

[14] Bohley P (1988). Proteolysis in hepatocytes. Adv. Clin. Enzym.

[15] Del Nery E, Juliano MA, Meldan M, Svendsen I, Scharfstein J, Walmsley A, Juliano L (1997). Characterization of the substrate specificity of the major cysteine protease (cruzipain) from *Trypanosoma cruzi* using a portion-mixing combinatorial library and fluorogenic peptides. Biochem. J.

[16] Stempin C, Giordanengo L, Gea S, Cerban F (2002). Alternative activation and increase of *Trypanosoma cruzi* survival in murine macrophages stimulated by cruzipain, a parasite antigen. J. Leukoc. Biol.

[17] Calabrese KS, Bauer PG, Lagrange PH, Goncalves da Costa SC (1992). *Trypanosoma cruzi* infection in immunosuppressed mice. Immunol. Lett.

[18] Soeiro MNC, Silva Filho FC, Meirelles MNL (1995). Alterations in the surface charge of heart muscle cells during interaction with *Trypanosoma cruzi*. Cell Biophys.

[19] Kausalya S, Malla N, Ganguly NK, Mahajan RC (1993). *Leishmania donovani*: *in vitro* evidence of hepatocyte damage by Kupffer cells and immigrant macrophages in a murine model. Exp. Parasitol.

[20] El Hag IA, Hashim FA, El Tourm IA, Homeida M (1994). Liver morphology and function in visceral leishmaniasis (Kala-azar). J. Clin. Pathol.

[21] Winwood PJ, Schuppan D, Iredale JP, Kawser CA (1995). Kupffer cell-derived 95-kDa type IV collagenase/gelatinase B: characterization and expression in cultured cells. Hepatology.

[22] Lindsey M, Wedin K, Brown MD, Keller C, Evans AJ, Smolen J, Burns AR, Rossen RD, Michael L, Entman M (2001). Matrix-dependent mechanism of neutrophil-mediated release and activation of matrix metalloproteinase 9 in myocardial ischemia/reperfusion. Circulation.

[23] Hijova E (2005). Matrix metalloproteinases: their biological functions and clinical implications. Bratisl. Lek. Listy.

[24] Burleigh BA, Woolsey AM (2002). Cell signalling and *Trypanosoma cruzi* invasion. Cell Microbiol.

